# Addition of lysophospholipids with large head groups to cells inhibits Shiga toxin binding

**DOI:** 10.1038/srep30336

**Published:** 2016-07-26

**Authors:** Ieva Ailte, Anne Berit Dyve Lingelem, Simona Kavaliauskiene, Jonas Bergan, Audun Sverre Kvalvaag, Anne-Grethe Myrann, Tore Skotland, Kirsten Sandvig

**Affiliations:** 1 Centre for Cancer Biomedicine, Faculty of Medicine, University of Oslo, Oslo, Norway; 2 Department of Molecular Cell Biology, Institute for Cancer Research, Oslo University Hospital, Montebello, Oslo, Norway; 3 Department of Biosciences, University of Oslo, Oslo, Norway; 4 Department of Research and Innovation, Østfold Hospital, Sarpsborg, Norway

## Abstract

Shiga toxin (Stx), an AB_5_ toxin, binds specifically to the neutral glycosphingolipid Gb3 at the cell surface before being transported into cells. We here demonstrate that addition of conical lysophospholipids (LPLs) with large head groups inhibit Stx binding to cells whereas LPLs with small head groups do not. Lysophosphatidylinositol (LPI 18:0), the most efficient LPL with the largest head group, was selected for in-depth investigations to study how the binding of Stx is regulated. We show that the inhibition of Stx binding by LPI is reversible and possibly regulated by cholesterol since addition of methyl-β-cyclodextrin (mβCD) reversed the ability of LPI to inhibit binding. LPI-induced inhibition of Stx binding is independent of signalling and membrane turnover as it occurs in fixed cells as well as after depletion of cellular ATP. Furthermore, data obtained with fluorescent membrane dyes suggest that LPI treatment has a direct effect on plasma membrane lipid packing with shift towards a liquid disordered phase in the outer leaflet, while lysophosphoethanolamine (LPE), which has a small head group, does not. In conclusion, our data show that cellular treatment with conical LPLs with large head groups changes intrinsic properties of the plasma membrane and modulates Stx binding to Gb3.

Membrane lipids are important regulators of basic cellular processes such as ligand binding, endocytosis and intracellular transport^1^. Glycerophospholipids and sphingolipids are common membrane lipids, with different classes and subtypes distributed asymmetrically between the two bilayers of the plasma membrane^2^,^3^. Cholesterol is an important regulator of plasma membrane fluidity and constitutes 30–50% of plasma membrane lipids^4^,^5^. Glycerophospholipids consist of a glycerol backbone and most often contain two hydrophobic fatty acid chains attached to *sn*-1 and *sn*-2 carbons and a polar head group attached to the *sn*-3 carbon through a phosphodiester linkage (Fig. 1). Lysophospholipids (LPLs) are glycerophospholipids with only one fatty acyl chain and are found in biological membranes at about 0.5–6% of total lipid weight^6^. Phospholipase A_2_ (PLA_2_) produces LPLs from glycerophospholipids by deacylating the fatty acyl at the *sn*-2 position (Fig. 1). The LPLs can be reacylated by lysophospho-acyltransferases. LPLs can have a conical shape when the surface area of the polar head group is larger than the corresponding area covered by the fatty acyl chain^7^. However, the conicity of different LPLs is strongly dependent on the size of the head group (Fig. 2) and, additionally, the saturation degree of the fatty acyl chain.

Despite their low abundancy, LPLs have several roles. Endogenous LPLs are important in membrane-shaping processes through the membrane phospholipid remodelling cycle, also known as Land’s cycle^8^. Exogenously added LPLs can be directly incorporated into cellular membranes^9^,^10^. Changes in LPL content and membrane curvature are important for vesicle fusion and fission processes that require membrane bending^3^,^11^,^12^.

Besides the structural roles, intracellular LPLs are lipid mediators in signalling^13^. Endogenously synthesized LPLs are secreted into physiological fluids such as blood, saliva and tears^14^,^15^. Circulating LPLs have been identified as ligands for a variety of G-protein coupled receptors (GPCRs)^16^, and activated GPCRs induce signalling cascades important for cell growth and differentiation, migration and cell-cell attachment, inflammation and immunity. Receptor-dependent and -independent roles of LPLs have been summarized in several comprehensive reviews^15^,^17^,^18^.

Increased levels of LPLs have been found in cancer patient blood and ascites, and a role in cancer pathophysiology has been established^19^,^20^,^21^. LPLs are therefore currently under investigation as cancer biomarkers^22^,^23^. The LPC analogue edelfosine alters membrane structure and induces apoptosis in cancer cells, and it has been tested as an anticancer drug, but showed too low efficacy and high toxicity in clinical trials^24^,^25^.

Recently, we published that exogenous addition of the conical lysophosphatidylinositol (LPI), but not phosphatidylinositol, protects cells against Shiga toxin (Stx) by inhibiting the binding of the toxin^26^. Whether this was due to a specific effect of LPI or if a similar effect could be obtained with other LPLs, was not tested at that time. In this study we have investigated whether various LPLs affect binding of protein toxins and explored the mechanistic details of how LPI affects Stx binding.

Stx belongs to an AB_5_ toxin family and consists of one enzymatically active moiety (A) and a binding pentamer (B_5_). Stx binds to the glycosphingolipid Gb3 at the surface of the plasma membrane, is internalized and sorted retrogradely towards the ER, from where the cytotoxic A moiety is translocated to the cytosol and inhibits protein synthesis, leading to cell death^1^. Each B unit has three binding sites with varying affinities for Gb3, providing up to 15 possible interactions between Stx and the carbohydrate moiety of the receptor^27^,^28^. The affinity of one interaction is at a millimolar scale, whereas multivalency leads to a very strong interaction between Stx and Gb3 receptor analogues, measured *in vitro*^29^,^30^. Multivalent binding at the plasma membrane is possible due to the mobility of glycolipids in the lipid bilayer, and clustering of receptors is important for optimally spaced multivalent binding^31^.

Figure 2 summarizes major classes of LPLs that were investigated in this study. The polar head group defines the class of the lysophospholipid while the fatty acyl chain length and saturation degree expands the molecular variety within each class. Here we demonstrate that insertion of LPLs with a large head group and a saturated fatty acyl chain affects membrane properties and the ability of Stx to bind to cells.

## Results

### Treatment with LPLs with large head groups can prevent cell binding and toxicity of Stx

Our earlier studies have revealed that LPI is able to protect cells against Stx by inhibiting binding of Stx to cells^26^. To investigate whether this inhibition was specific for LPI or whether also other LPLs could affect Stx binding, we treated cells with different classes of LPLs and measured Stx binding to HEp-2 cells. All substances tested are listed in Table 1. As shown in Fig. 3a, LPLs with large head groups (LPI, LPS, LPC) and edelfosine have an inhibitory effect on Stx binding in a concentration-dependent manner, while LPLs with small head groups (LPE and LPA) do not have such an effect. The LPC analogue edelfosine was included in this study to investigate if any of the effects observed here are due to enzymatic conversion of LPLs. Edelfosine has a similar shape as LPC, but edelfosine contains an ether-linked methyl group in the *sn*-2 position instead of a hydroxyl group, and an ether linked C18:0 in the *sn*-1 position, making it metabolically stable. LPC 18:0 and edelfosine C18:0 gave very similar effect on Stx binding, as shown in Fig. 3a.

To investigate a possible importance of the fatty acyl chain length in LPI-mediated effects, we compared the inhibitory effects of synthetic pure LPI 18:0, LPI 16:0 and LPI from bovine liver containing mostly 18:0 fatty acyl chains (further on called LPI) on Stx binding. Indeed we found that LPI and LPI 18:0 give a stronger reduction in Stx binding than LPI 16:0, suggesting that the inhibitory effect of LPI also depends on the length of the fatty acyl chain (Fig. 3b,c).

Data presented in Fig. 3a–c indicated that the geometry of the lipids might determine the potency of various LPLs to inhibit the binding of Stx. An unsaturated carbon bond in the fatty acyl chain induces a kink and changes the shape and packing abilities of the lipid molecules. We therefore compared the effects of saturated LPC 18:0 with unsaturated LPC 18:1 (Δ9) and found that LPC 18:1 has no effect on Stx binding, whereas LPC 18:0 strongly inhibits binding (Fig. 3d).

**Figure 2:**
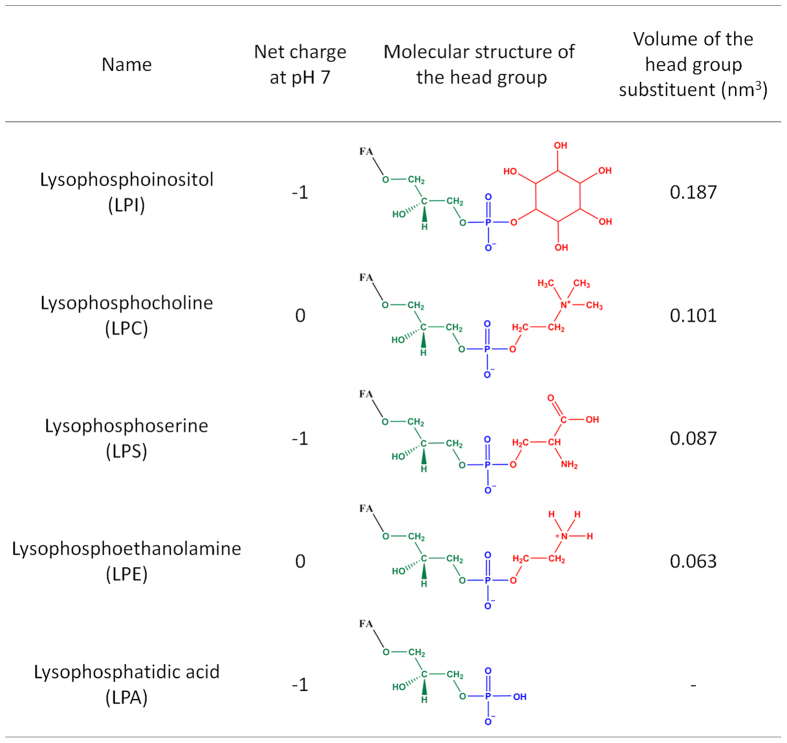
Names and molecular structures of LPLs used in this study. The lysophospholipids are sorted in order according to the head group size from largest to smallest: LPI > LPC > LPS > LPE ≫ LPA. The glycerol backbone is indicated in green, the phosphodiester bond in blue and the head group in red. The volumes (nm^3^) for the head group substituents (red) are taken from Lundbaek and Andersen^7^. For lysophosphatidyl-lipids, FA is a fatty acyl attached to the glycerol backbone through an ester bond.

**Figure 3:**
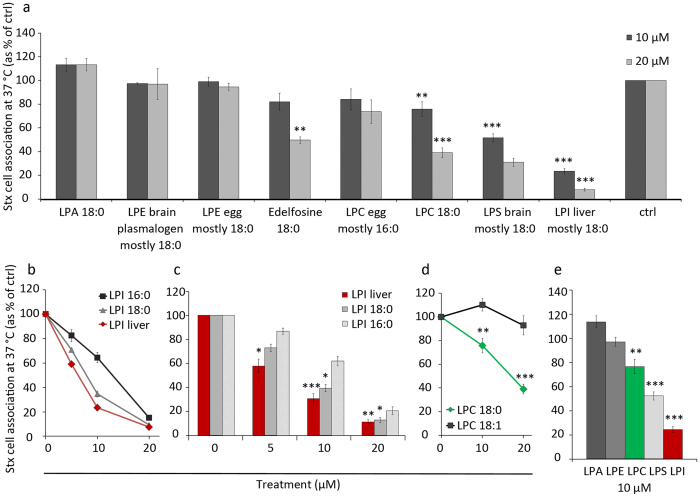
LPL treatment effects on Stx cell association. HEp-2 cells were pretreated with LPLs or other substances for 30 min in HEPES-buffered medium without FBS for 30 min at 37 °C, prior to incubation with ^125^I-Stx1m for 20 min at 37 °C. Cells were washed, lysed and the radioactivity was measured. All panels show quantification data of cell-associated ^125^I-Stx1m, expressed as % of control. **(a)** Cells were pretreated with 10 or 20 μM LPLs before incubation with ^125^I-Stx1m (mean ± SEM; *n* ≥ 3, except for 20 μM LPS brain and 20 μM LPE brain plasmalogen showing mean ± STD; *n* = 2). **(b)** A representative figure for one out of five independent experiments, showing amounts of cell-associated ^125^I-Stx1m when treated with LPI (bovine liver LPI, mostly 18:0), pure LPI 18:0 and pure LPI 16:0 at increasing concentrations (mean ± STD, duplicates). **(c)** Quantification of cell-associated ^125^I-Stx1m after treatment with LPI (bovine liver, mostly 18:0), LPI 18:0 and LPI 16:0 at increasing concentrations (mean ± SEM; *n* ≥ 5). **(d)** Quantification of cell-associated ^125^I-Stx1m after treatment with LPC 18:0 and LPC 18:1 at 10 and 20 μM (mean ± SEM; *n* = 3). **(e)** Quantification of cell-associated ^125^I-Stx1m upon treatment with various LPLs with acyl chains containing mostly 18:0 or pure 18:0 (mean ± SEM; *n* ≥ 3).

To compare the effect of the head group size on Stx binding, only LPLs with chain length 18:0 or mostly 18:0 were presented in a separate figure. The LPLs were ordered according to the inhibitory effect on Stx binding (Fig. 3e). The degree of inhibition corresponds nicely with the size of the LPL head groups, as shown in Fig. 2. There is almost no inhibitory effect of the LPLs with the smallest head groups and approximate 80% inhibition with the largest head group. An equivalent treatment of HeLa cells with LPLs gave very similar results to that shown for HEp-2 cells (Supplementary Fig. S1).

### LPL effects on toxin cell association, transport and toxicity

**Figure 4:**
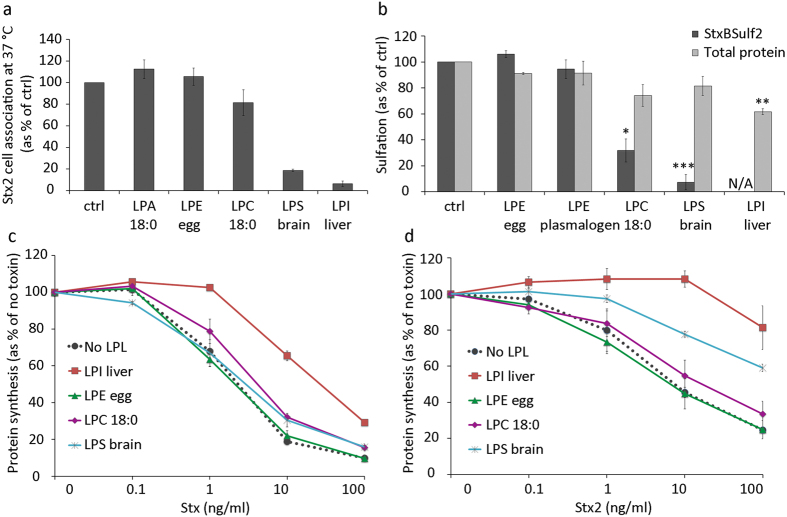
LPL effects on toxin cell association, transport and toxicity. **(a)** HEp-2 cells were pretreated with 10 μM LPLs with acyl chains consisting of pure or mostly C18:0 for 30 min, prior to incubation with ^125^I-Stx2 for 20 min at 37 °C. Cells were washed, lysed and the radioactivity was measured. Cell-associated ^125^I-Stx2 is expressed as % of control (mean ± deviation; *n* = 2). **(b)** Sulfation assay to measure the amounts of StxBSulf2 transported to the Golgi apparatus where the modified toxin is sulfated. HEp-2 cells were pre-incubated with ^35^SO_4_^2−^ for 1.5 h and then incubated with 10 μM LPLs with acyl chains consisting of pure or mostly C18:0 for 30 min, followed by addition of 2 μg/ml StxBS2 for 1.5 h. Shiga toxin was immunoprecipitated from cell lysates and radioactivity was counted. Results are shown as % of ctrl (mean ± SEM; *n* = 3). N/A stands for not applicable (below detection limit). **(c,d)** Toxicity assay with Shiga holotoxin (Stx) and Stx2. Cells were pretreated with 5 μM LPLs with acyl chains consisting of pure or mostly C18:0 for 30 min, followed by incubation with tenfold serial toxin dilutions for 4 h in leucine-free medium. Protein synthesis was measured and results are shown as % of no toxin (mean ± deviation; *n* = 2 for each of the toxins).

Shiga toxin 2 (Stx2) is produced by *Escherichia coli* (*E. coli*) and is genetically and immunologically distinct from Stx (produced by *Shigella dysenteriae*) and Shiga toxin 1 (produced by *E. coli*), which is almost identical to Stx, but all these toxins share similar structure and enzymatic function^32^,^33^. However, Stx2 is more often associated with serious disease than Stx1^33^. We decided to investigate whether treatment with LPLs could inhibit binding and cytotoxicity also of Stx2. As shown in Fig. 4a, treatment with conical LPLs (10 μM) with large head groups (LPI with mostly C18:0 and LPS with mostly C18:0) strongly inhibited cell association of Stx2, while LPLs with small headgroups (LPA 18:0 and LPE with mostly C18:0) did not. Very similar results were obtained when Stx2 binding was performed on ice (not shown).

We also investigated the effect of LPL treatment on intracellular transport of Stx and toxic activity on cells, and our data show that the effect of different LPLs on these events corresponds to the LPL effect on binding. As shown in Fig. 4b, Stx transport to the Golgi was completely inhibited by LPI and strongly reduced by LPS and LPC, measured as sulfation of a modified Stx binding moiety with two sulfation sites attached (StxBSulf2). LPI provided a strong protection against Stx and Stx2 (Fig. 4c,d).

### The LPL-induced inhibition of Stx binding is independent of intracellular signalling and membrane turnover

**Figure 5:**
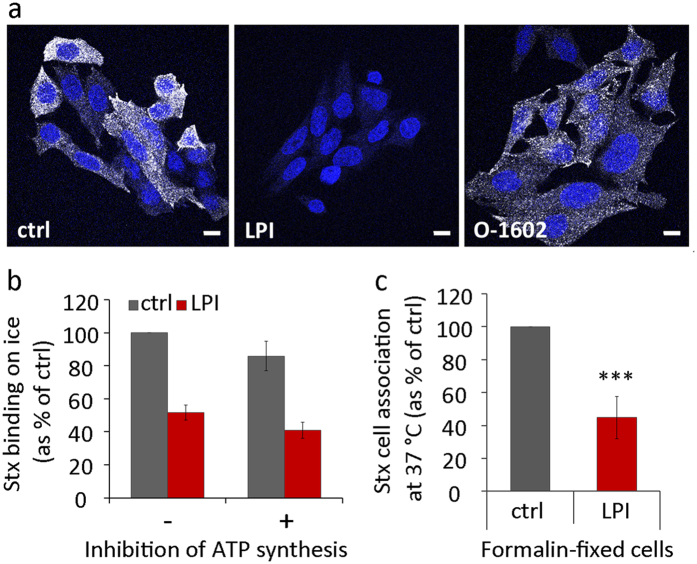
LPI effects on Stx binding are independent of signalling and ATP. **(a)** HEp-2 cells were pretreated with LPI (10 μM) or GPR55 agonist O-1602 (10 μM) for 10 min prior to addition of Stx1m (100 ng/ml) for 30 min at 37 °C. Cells were washed, fixed, permeabilized and blocked in 5% FBS for 30 min prior to incubation with primary and secondary antibodies. Confocal microscopy images show Stx staining in white and nuclei in blue; scale bar 10 μm. **(b)** Cells were pretreated with or without 2-deoxy-D-glucose (50 mM) and NaN_3_ (10 mM) for 1 h to deplete cellular ATP before 10 μM LPI was added and incubation was continued for 30 min. Cells were then cooled down for 10 min and ^125^I-Stx1m was added for 20 min on ice. Cells were washed, lysed and the radioactivity signal was counted. Results are presented as % of control (mean ± STD; *n* = 3 for ctrl and *n* = 2 for LPI). **(c)** Cells were washed with HEPES-buffered medium to remove serum proteins and fixed with 10% formalin solution for 30 min. The fixed cells were treated with 10 μM LPI for 30 min at 37 °C and then incubated with ^125^I-Stx1m for 20 min at 37 °C. Cell-associated ^125^I-Stx1m was counted and presented as % of control (mean ± SEM; *n* = 3).

LPLs are bioactive signalling molecules that can bind to specific GPCRs at the plasma membrane and activate cell signalling cascades. GPR55 is a receptor for LPI and O-1602 is an agonist^16^,^34^. To investigate if the effects observed here are dependent on GPR55 signalling, we tested whether the binding of Stx could be inhibited by treating cells with O-1602. This was not the case since confocal microscopy images showed persistent Stx uptake upon treatment with the agonist, but strong inhibition of binding with LPI (Fig. 5a). In addition, other lysophospholipids (LPC and LPS) that are known to bind to their own specific GPCR^34^ also inhibit binding of Stx, as shown in Fig. 3a.

To test further whether cell signalling is required for the inhibition of Stx binding, we depleted cellular ATP prior to the LPI treatment. The effects of LPI on Stx binding on ice were not affected after inhibition of ATP synthesis (Fig. 5b), supporting the idea that the effects are signalling independent and also not due to a change in membrane turnover.

Finally, we also tested if LPI would prevent Stx binding on formalin-fixed cells. We performed a chemical cell fixation in 10% formalin solution that crosslinks amine and amide groups of proteins in cells^35^, while membrane lipids stay less affected and retain some mobility^36^. Cell fixation itself reduced Stx binding to cells by 2-fold, but we could still see the same degree of inhibition upon LPI treatment (Fig. 5c) as in live cells, and no inhibition of Stx binding upon treatment with 10 μM LPA (not shown). In summary, these results clearly show that LPI-induced inhibition of Stx binding is independent of receptor signalling and ATP, indicating that LPI directly affects Gb3 behaviour at the plasma membrane.

### Characterization of LPI-induced inhibition of Stx binding to cells

**Figure 6:**
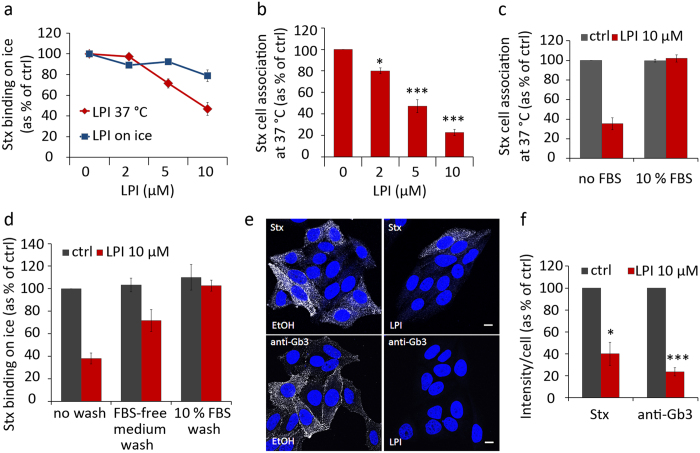
Characterization of LPI-induced inhibition of Stx binding to Gb3. **(a)** HEp-2 cells were pretreated with increasing concentrations of LPI for 30 min at 37 °C or on ice, prior to cooling down and incubation with ^125^I-Stx1m for 30 min on ice. Cells were washed, lysed and the radioactive signal was measured. Results are presented as % of control (mean ± SEM; *n* = 3). **(b)** Cells were pretreated with increasing concentrations of LPI for 30 min at 37 °C, prior to incubation with ^125^I-Stx1m for 20 min at 37 °C. Cells were washed, lysed and the radioactive signal was measured, results are presented as % of control (mean ± SEM; *n* = 3). **(c)** Cells were pretreated with 10 μM LPI for 30 min in absence or presence of 10% FBS prior to incubation with ^125^I-Stx1m for 20 min at 37 °C. The quantification of radioactivity measurements is shown, presented as % of control (mean ± SEM; *n* = 3). **(d)** Cells were incubated with 10 μM LPI for 30 min and then washed once at 37 °C with HEPES-buffered cell growth medium with or without 10% FBS, prior to cooling down on ice for 10 min and incubation with ^125^I-Stx1m on ice for 20 min. The quantification of radioactivity measurements is shown, presented as % of control (mean ± STD; *n* = 2). **(e)** Cells were pretreated with 10 μM LPI for 30 min prior to addition of Stx1m (100 ng/ml) or anti-Gb3 antibody and incubated for 20 min at 37 °C. Cells were washed, fixed, permeabilized and blocked in 5% FBS for 1 hr prior to incubation with primary (Stx samples only) and secondary antibodies. Confocal microscopy images show Stx or anti-Gb3 antibody staining in white and DAPI nuclear staining in blue; scale bar 10 μm. **(f)** Quantification of intensity signals of Stx and anti-Gb3 antibodies from confocal images, expressed as % of control (mean ± SEM; *n* = 3).

Of all the LPLs tested, both the bovine liver LPI (mostly 18:0) and the synthetic pure LPI 18:0 gave the strongest reduction in Stx binding. We decided to study the ability of LPI to inhibit Stx in more detail and used LPI from bovine liver (called LPI in the text and figures) for this purpose. First we tested for how long cells had to be incubated with LPI (10 μM) to obtain inhibition of binding. We observed a strong inhibition already after 10 min and maximal inhibition after 30 min (not shown). Next we tested whether LPI had to be incubated with the cells at 37 °C to inhibit binding of Stx, and as shown in Fig. 6a, this was indeed the case. Pretreatment with LPI on ice gave only a slight effect on Stx binding on ice compared to more than 50% reduction in Stx binding on ice when lipid treatment was performed at 37 °C (Fig. 6a). This is in agreement with the idea that LPI has to be inserted into the membrane for its action.

We extended our studies of the concentration dependent effect of LPI on Stx cell association at 37 °C, and the data reveal that even 2 μM LPI has an inhibitory effect (Fig. 6b). It should be mentioned that LPI concentrations higher than 20 μM were avoided as it can lead to cell rounding and detachment, and is close to the critical micelle forming concentration of 30 μM for LPI 18:0^37^.

LPI is transported in blood, bound to albumin^34^. We therefore pretreated cells with 10 μM LPI in the presence of 10% fetal bovine serum (FBS) to see if we could abolish the LPI-mediated effect on Stx binding. And indeed, there was no reduction in Stx binding when cells were pretreated with LPI in the presence of FBS (Fig. 6c). In agreement with this finding, addition of FBS-containing medium to LPI-treated cells rapidly restored the ability of the cells to bind Stx (Fig. 6d). Even just washing the cells with HEPES-buffered cell growth medium without FBS was sufficient to diminish the effect of LPI on Stx binding, indicating that there is an equilibrium between the aqueous LPI and the LPI inserted into the membrane (Fig. 6d).

Interestingly, LPI treatment not only inhibited binding of Stx, but also binding of an IgM antibody against Gb3 (Fig. 6e). The fluorescence intensity signals of Stx and Gb3 antibody staining on confocal images were quantified, and we observed a significant reduction for both ligands (Fig. 6f).

**Figure 7:**
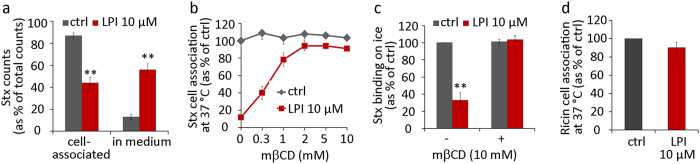
LPI releases prebound Stx; the inhibitory effect of LPI on Stx binding is reversed by mβCD; LPI has almost no inhibitory effect on binding of ricin. **(a)** HEp-2 cells were incubated with ^125^I-Stx1m on ice for 30 min, washed and treated with 10 μM LPI for 20 min at 37 °C. Cell medium was collected, cells were lysed and the radioactivity signal was counted. The quantification of ^125^I-Stx1m counts is expressed as % of total counts (mean ± SEM; *n* = 3). **(b)** Cells were pretreated with 10 μM LPI for 30 min and then increasing concentrations of mβCD were added and treatment continued for 15 min. Cells were then incubated with ^125^I-Stx1m for 20 min at 37 °C and cell-associated ^125^I-Stx1m was counted, quantification results presented as % of control (mean ± SEM; *n* = 3). **(c)** Cells were pretreated with 10 μM LPI for 30 min and then 10 mM mβCD was added and treatment continued for 15 min. Cells were then incubated with ^125^I-Stx1m for 20 min on ice and cell bound ^125^I-Stx1m was counted, quantification results presented as % of control (mean ± SEM; *n* = 3). **(d)** Cells were pretreated with 10 μM LPI for 30 min at 37 °C, prior to incubation with ^125^I-ricin (20 min 37 °C). After the treatment, cells were washed, lysed and the radioactive signal was measured. Quantification data are presented as % of control (mean ± SEM; *n* = 6).

To investigate whether LPI can release Stx already bound to cells, we prebound Stx to cells on ice and then added LPI at 37 °C. Remarkably, LPI was able to induce dissociation of prebound Stx from the cell surface. Cell-associated Stx was reduced by more than 50% after 20 min incubation with LPI at 37 °C (Fig. 7a).

### LPI effect on Stx binding is reversed by methyl-β-cyclodextrin (mβCD)

Cholesterol is an important regulator of membrane organization in general, and it can also interact with Gb3^38^,^39^ thereby changing the orientation of the carbohydrate moiety relative to the membrane. MβCD is commonly used to extract membrane cholesterol and is reported to be cholesterol-specific at concentrations below 10 mM, with only negligible binding of other lipids at such low concentrations^40^. We tested whether the reduced binding caused by LPI could be restored after cellular treatment with mβCD. In control cells, mβCD treatment for 15 min at concentrations 0.3–10 mM did not affect Stx binding on ice or Stx cell association after 20 min incubation at 37 °C (Fig. 7b,c). In contrast, addition of mβCD after cell pretreatment with LPI rescued both the Stx binding on ice and cell-associated Stx at 37 °C (Fig. 7b,c).

### LPI has almost no effect on binding of ricin

To address whether binding of ligands to other receptors is affected by LPI treatment, we investigated the binding of ricin which is an AB class toxin. Ricin has only one binding subunit that binds to various membrane glycolipids and glycoproteins with a terminal galactose. The cell association of ricin was only slightly affected by 10 μM LPI treatment (reduction by 10%), as shown in Fig. 7d. Other substances tested gave similar results (LPC, LPS, LPE, lysoplasmalogen LPE and edelfosine; data not shown).

### LPL treatment and membrane lipid packing

Lipid packing is one of the key physicochemical properties of biological membranes. Multiple environment-sensitive dyes, which change their spectroscopic properties in response to environment parameters, such as polarity, hydration, viscosity, etc., have been developed and used to study lipid packing in both artificial and biological membranes^41^. Quantitative assessment of lipid packing is achieved by measuring the shift in the dye emission profile between liquid ordered (Lo) and liquid disordered (Ld) phases, giving a relative index called generalized polarization (GP)^42^,^43^. The shift in emission is due to the polarization state of the dye which is affected by accessibility of the dye to water molecules.

To study the effects of LPI and other LPLs on the plasma membrane lipid packing, we used a recently developed environment-sensitive probe NR12S based on the Nile Red fluorophore used for lipophilic staining. In line with other environment-sensitive probes, such as Laurdan and di-ANEPPDHQ^43^, NR12S shows a blue-shifted emission in Lo phase vesicles and a red-shifted emission in Ld phase vesicles^44^. Important key features of NR12S are its ratiometric response to lipid packing (Lo vs. Ld phase) and insensitivity to surface charge, selective localization to the outer leaflet of the plasma membrane with low internalization rate and a high fluorescence quantum yield^44^, which makes it suitable for studying plasma membrane properties in live cells. Cholesterol extraction has been shown to red-shift the emission spectrum of NR12S, indicating a decrease in lipid order at the outer plasma membrane leaflet^44^,^45^ and thus cholesterol depletion by mβCD was used as a positive control for a shift towards Ld phase in our study. Cell treatment with 0.2% ethanol was used as a reference and gave no notable effect on lipid packing when compared to medium only.

**Figure 8:**
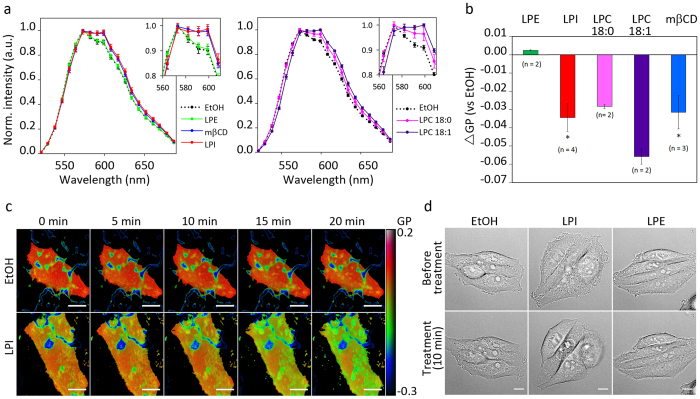
LPL treatment changes plasma membrane lipid packing. **(a)** HEp-2 cells were treated with 10 μM LPI, LPE, LPC 18:0, LPC 18:1 or 0.2% ethanol (EtOH) for 20 min, or with 5 mM mβCD for 45 min, prior to staining with 10 nM NR12S. Spectral imaging was started 7 min after the addition of NR12S and continued for up to 15 min. The analysis of the spectra was performed on at least 20 manually selected regions of the plasma membrane for each condition. The panel to the left shows the data from one of three independent experiments with LPI, LPE and mβCD and the panel to the right shows data from one of two independent experiments with LPC 18:0 and LPC 18:1 (mean ± STD). **(b)** The average GP value for each selection was quantified as described in materials and methods, and the figure shows differences in GP values between the EtOH and the treated samples (mean ± SEM; *n* ≥ 2). **(c)** Pseudo-coloured TIRF images of the basal plasma membrane following cell treatment with 0.2% EtOH or 10 μM LPI. The dark blue structures are the coating of the Lab-Tek dish stained by NR12S. The colour scale for GP values is shown on the right; scale bar 10 μm. **(d)** Differential interference contrast images showing morphology of HEp-2 cells, taken 10 min prior to and 10 min after addition of 0.2% EtOH, 10 μM LPI or 10 μM LPE; scale bar 10 μm.

First, we performed spectral imaging with a laser scanning microscope, where the whole emission spectrum of the NR12S was registered, to look for any effects on lipid packing in the plasma membrane caused by various treatments in live HEp-2 cells. We observed a clear red-shift in the emission spectrum of the dye when cells were treated with 10 μM LPI for 20 min prior to staining with NR12S (Fig. 8a). The analysis of the spectrum was performed on selected regions of the plasma membrane free for filopodia and cell-cell contacts. This was done in order to make sure that the effects we measure are directly mediated by LPI, and not due to changes in cell morphology. Quantitative analysis of lipid packing was performed by calculating the change in generalized polarization value (ΔGP) compared to ethanol. Treatment with 10 μM LPI gave a significant decrease in the GP, while 10 μM LPE had no effect on the GP value (Fig. 8b).

Since we had found that treatment with LPC 18:1 in contrast to LPC 18:0 did not affect binding of Stx, we investigated the effect of both LPC 18:1 and LPC 18:0 on lipid packing. We show that cell treatment with LPC 18:1 gives a strong red-shift within the outer leaflet, demonstrating that, even thought it does not inhibit Stx binding (Fig. 3d), it becomes incorporated in the plasma membrane (Fig. 8a,b). This further strengthens the idea that it is membrane insertion of conical LPLs with a large head group and a saturated fatty acyl chain that inhibits binding of Stx.

To study the dynamics of lipid packing upon LPI treatment, we performed TIRF time-lapse imaging where the basal part of the plasma membrane was imaged every 5 min for 20 min. Cells were pre-stained with NR12S before addition of LPI or ethanol, and we observed a clear reduction in GP value (Fig. 8c). Furthermore, we investigated the effect of addition of 10 μM LPI on the whole cell morphology by live cell imaging using wide-field microscopy and observed that HEp-2 cells rapidly obtained a more rounded surface and had less filopodia upon LPI treatment (Fig. 8d).

## Discussion

We here report that insertion of conical LPLs with large head groups inhibits binding of Stx and Stx2 to cells and that LPI even can induce dissociation of cell-bound Stx. A number of studies have revealed that binding of Stx to its receptor Gb3 is dependent not only on the structure of the receptor itself, but also on the membrane environment^38^,^39^,^46^. Physical properties of the plasma membrane are regulated by the lipid composition and temperature, and in this study we have investigated effects of adding LPLs with different head groups and fatty acyl chains. Earlier studies on HeLa cells have revealed that radioactively labelled LPI rapidly gets inserted into the outer leaflet of the plasma membrane^10^ and that the rate of translocation to the inner leaflet is slow^3^. Similar observations have been made with radioactively labelled LPC on human erythrocytes^9^. The rapid insertion, lack of transmembrane translocation and large head group to acyl chain ratio forms the basis for the ability of LPLs with large head groups to induce the formation of positive curvature^47^. Such a change in membrane curvature is in agreement with the morphological alterations we observe in HEp-2 cells incubated with LPI. Similar morphological cell-shape changes have been reported in CHO-K1 cells upon incubation with LPI^48^. In contrast, we observed that LPE, which has a small head group, does not induce any changes in cell morphology, possibly due to the smaller head group to fatty acyl chain area ratio that gives less impact on lipid packing in the outer leaflet of the plasma membrane. An alternative explanation is a more uniform distribution of LPLs with small head groups between the two leaflets due to a more rapid translocation to the inner leaflet. However, this seems less likely due to short term experiments here used and experiments performed on ATP depleted and also fixed cells.

As demonstrated here, LPLs can affect lipid packing of the outer leaflet of the plasma membrane with a change towards a more liquid disordered phase, shown by the shift in the fluorescence of the NR12S probe which is predicted to be localized in the outer leaflet of the plasma membrane^44^. Since the fluorescence of the NR12S has been shown to be independent of the surface charge of the membrane^44^, it should specifically respond to changed packing of the fatty acyl chains rather than to alterations at the head groups of the lipids. It is therefore tempting to hypothesize that insertion of the conical LPI and LPC 18:0 reduces the packing of the fatty acid groups by introducing more space between the adjacent acyl chains, while there might be an opposite effect at the surface of the lipid layer with increased packing of the head groups. Consequently, lipid packing may contribute to regulate the presentation of the carbohydrate epitope and/or the lateral diffusion of Gb3 in the membrane, as such dependency has been shown for other receptors^49^.

Importantly, although both LPI and mβCD give a shift towards liquid disordered phase in the membrane, only LPI reduced Stx binding to the cells, suggesting that it is not the reduction in the lipid packing as such (at least not at the level of the fatty acyl chains) that leads to reduced Gb3 availability for binding. An interesting possibility is that the treatment of cells with LPI or LPC 18:0 and the observed increase in the disordered phase might facilitate interaction of Gb3 with cholesterol, bend the carbohydrate part and thereby inhibit Stx binding^38^,^39^. The reduced binding could then be restored by extraction of cholesterol. Furthermore, it has been reported that increased membrane disorder in erythrocyte membranes obtained by extraction of cholesterol with mβCD can increase flip-flop of exogenously added LPC from the outer to the inner leaflet^50^, whereas addition of cholesterol will decrease the movement of LPC^51^. Thus, an alternative or additional explanation for the ability of mβCD to restore binding of Stx is that it promotes movement of LPI to the inner leaflet, thereby reducing the amount of LPI in the outer leaflet.

The finding that LPC 18:1 gives a strong increase in the lipid disordered phase, but has no effect on binding of Stx strengthens the idea that it is the shape of the lipid that is important for inhibition of toxin binding. Also, if conical LPLs increase the crowding of head groups, this might lead to reduced Gb3 diffusion and thus inhibit multivalent binding of Stx to the Gb3. The requirement for multivalent binding and also for specific carbohydrate conformations to fit with the binding sites of Stx might explain why Stx binding is inhibited by LPI treatment, whereas there is essentially no effect on binding of the AB toxin ricin.

In summary, our study reveals that it is the geometrical shape of the LPL that determines its efficiency in preventing cellular binding of Stx. The potency depends on both the size of the head group and the length and saturation status of the fatty acid chain. Our data suggest that treatment with conical LPLs with large head groups both changes the physicochemical properties of the plasma membrane and leads to an altered Gb3 receptor conformation and/or distribution.

## Materials and Methods

### Reagents and antibodies

All chemicals were purchased from Sigma-Aldrich (St. Louis, MO, USA) unless stated otherwise. Shiga toxin 1-mutant (Stx1m) was a kind gift from Dr A. D. O’Brien (Uniformed Services University of the Health Sciences, Bethesda, MD, USA) and was purified as previously described^52^. Purified Shiga holotoxin was a gift from Dr J.E. Brown (USAMRIID, Fort Detrick, MD, U.S.A.) and Dr L. Kozlov (Academy of Science of Russia, Moscow, Russia). Stx2^26^ and StxBSulf2^53^ were purified as previously described. Ricin holotoxin was purchased from Sigma-Aldrich. The iodine-125 radionuclide (^125^I) for radioactive protein labelling (NEZ033A010MC) was from Perkin Elmer (Waltham, MA, USA). Methanol-free 16% (w/v) paraformaldehyde solution (cat. no. 18814-20) for chemical cell fixation was from Polysciences (Warrington, PA, USA). The GPR55 agonist O-1602 (cat. no. 2797) was purchased from Tocris Bioscience (Bristol, UK).

Primary antibodies: monoclonal IgG anti-Stx1 (3C10, Toxin Technology, Inc., FL, USA) and monoclonal anti-Gb3 (anti-human CD77) antibody IgM (cat. no. 551352, BD Pharmingen, NJ, USA). Secondary antibodies: IgG Alexa Fluor 488 (cat. no. 715-545-150) and IgM Alexa Fluor 488 (cat. no. 715-545-140), both by Jackson Immuno Research (West Grove, PA, USA). Prolong Gold Antifade Reagent with DAPI for nuclear staining and mounting was from ThermoFisher Scientific (Waltham, MA, USA). The environment-sensitive probe NR12S was a kind gift from Prof. A. Klymchenko (University of Strasbourg, Strasbourg, France).

### Lysophospholipids (LPLs)

LPLs were purchased from Avanti Polar Lipids (Alabaster, AL, USA) if not otherwise stated. All lipids applied in this study are presented in Table 1 in an alphabetic order. The lipids were purchased in a powder form and dissolved in ethanol : H_2_O (1 : 1) to obtain a main stock concentration of 2.5 mM and stored at −20 °C. As control treatment we used ethanol corresponding to the concentration of ethanol in the LPL volume added, e.g. for treatment with 10 and 20 μM LPLs, 0.2% and 0.4% ethanol was used for control, respectively. Molecular structures of LPLs presented in Fig. 2 were drawn in ChemDraw (Perkin Elmer).

### Cell lines

HEp-2 cells (ATCC: CCL-23, 1994) and HeLa (Institute Curie, Paris, France, 2002) were grown in a humidified 5% CO_2_ atmosphere at 37 °C and maintained in Dulbecco’s modified Eagle’s medium with high glucose (DMEM; D0819, Sigma-Aldrich) supplemented with 10% FBS and 100 U/ml penicillin and 100 U/ml streptomycin. Cells were seeded at concentrations of 5 × 10^4^ cells/well in 4 or 24 well plates of type Nunc by ThermoFisher Scientific or Falcon by BD Biosciences (Franklin Lakes, NJ, USA), 3 × 10^4^ cells/well in Nunc Lab-Tek II chambered coverglass dishes (ThermoFisher Scientific) or 1 × 10^5^ cells/well in 35 mm MatTek glass bottom dishes (MatTek Corporation, Ashland, MA, USA) one day prior to experiments. The growth medium was changed to serum-free HEPES-buffered cell growth medium supplemented with Glutamax (cat. no. 35050-061, ThermoFisher Scientific) at the beginning of the experiments, unless stated otherwise.

### Cell binding and uptake assays with ^125^I- labelled ligands

The enzymatically inactive mutant of Shiga toxin, called Stx1m in figures and legends, but called Stx in the text for simplicity, Stx2 and ricin were labelled with ^125^I using Pierce Iodination Reagent IODO-GEN (cat. no. 28600, ThermoFisher Scientific) according to the manufacturer’s protocol. The yield of iodine incorporation varied slightly from batch to batch and was around 25 000 cpm/ng for ^125^I-Stx1m, 220 000 cpm/ng ^125^I-Stx2, 30 000 cpm/ng ^125^I-ricin right after the labelling. Cells were seeded in 24 well plates at standard conditions and treated with different concentrations of LPLs in 200 μl HEPES-buffered cell growth medium for different periods of time at 37 °C or on ice as indicated in figure legends. For cell binding at 37 °C, approximately 10 ng ^125^I-Stx1m/well, 10 ng ^125^I-Stx2/well or 10–25 ng ^125^I-ricin/well were added and then incubated for 20 min at 37 °C. For toxin binding on ice, cells were first cooled down on ice for 10 min and then incubated with the toxins for 30 min on ice. Cells were washed three times with ice-cold phosphate-buffered saline (PBS) and lysed in 0.1 M KOH. The radioactivity was measured using a LKB Wallac 1261 Multigamma counter (LKB Instruments, Victoria, Australia).

### Sulfation of StxBSulf2

HEp-2 cells were washed twice with sulfate-free medium (SFM), and incubated with 0.2 mCi/ml ^35^SO_4_^2−^ for 1.5 h at 37 °C in SFM, followed by incubation with 10 μM LPLs for 30 min at 37 °C and then 2 μg/ml StxBSulf2 for 1.5 h. Precipitation and quantification of sulfated StxBSulf2 was performed as previously described^53^.

### Toxicity assay

Cells were washed with leucine-free HEPES-buffered medium and pretreated with 5 μM or 10 μM LPLs for 30 min at 37 °C, prior to incubation with ten-fold serial dilutions of Shiga holotoxin or Stx2 in HEPES-buffered medium for 4 h at 37 °C. Then, the cells were washed and incubated with leucine-free HEPES-buffered medium containing 1 μCi/ml [^3^H]leucine (PerkinElmer, MA, USA) for 20 min at 37 °C. The proteins were precipitated with 5% (w/v) trichloroacetic acid (TCA), washed once with 5% (w/v) TCA, and dissolved in 0.1 M KOH. The amount of incorporated [^3^H]leucine was measured by β-counting. We observed some reduction in protein synthesis due to the lipid treatment with the most potent lipids at 10 μM when incubated for 4.5 h so we show the toxicity data from the experiment with 5 μM LPL.

### Immunofluorescence staining and confocal microscopy

HEp-2 cells were seeded out on coverslips in 4-well plates and grown for one day. First, the cells were pretreated with 10 μM LPI for 30 min at 37 °C and then incubated with 100 ng/ml Stx1m or Gb3 antibody (5 μg/ml) for 20 min at 37 °C. Cells were then washed and fixed with 3% methanol free EM grade PFA (Gb3 samples) or 10% formalin solution (Stx samples) for 5 min on ice and then 15 min at room temperature (RT), followed by permeabilization in 0.1% TritonX-100 for 2 min and blocking in 5% FBS in PBS for 1 h at RT. The Stx samples were incubated with primary antibody (4 μg/ml STX1-3C10) in 5% FBS in PBS for 1 h at RT followed by incubation with secondary antibodies IgG Alexa Fluor 488 1:500 (Stx samples) or IgM Alexa Fluor 488, 1:500 (Gb3 samples). Cells were then washed, fixed in 10% formalin solution and permeabilized in 0.1% Triton X-100 for 2 min. The samples were mounted in ProLong Gold with DAPI and investigated using a Zeiss LSM 710 or 780 laser scanning confocal microscope (Carl Zeiss MicroImaging, Jena, Germany) equipped with an Ar-Laser Multiline (458/488/514 nm), a DPSS-561 10 (561 nm), and a Laser diode 405–30 CW (405 nm). The objective used was a Zeiss Plan-Apochromat 63x/1.40 Oil DIC M27. Images were acquired using the ZEN 2010 software (Carl Zeiss MicroImaging). Fiji software^54^ was used for quantification of signal intensities and for image preparation.

### Spectral GP imaging on confocal microscope

HEp-2 cells were seeded in 8-well Lab-Tek chambered coverglass dishes at a density of 3 × 10^4^ cells/well one day before the experiment. The cells were washed twice with warm HEPES-buffered Live Cell Imaging Solution (cat. no. A14291DJ, ThermoFisher Scientific) and then incubated with 10 μM LPI, LPC 18:0, LPC 18:1, LPE or 0.2% ethanol or with Live Cell Imaging Solution alone for 20 min at 37 °C. As a positive control for an increase in membrane fluidity, cells were treated with 5 mM mβCD for 45 min at 37 °C, and then medium was replaced with fresh Live Cell Imaging Solution. To stain cell membranes with NR12S probe, NR12S was diluted in Live Cell Imaging Solution and immediately added to cells. After 7 min, the imaging was started and continued for 10–15 min resulting in 8–10 images taken per condition. The images were acquired using Zeiss LSM 780 confocal microscope equipped with a 63x objective, NA 1.4 and a 32-channeled GaAsP detector array. Laser light at 514 nm was used for the excitation of NR12S, and the fluorescence detection range was set between 521 and 687 nm with 8.7 nm intervals. The focus was set to equatorial plane of the cells slightly above the cover glass surface. The sample chamber was controlled for temperature (37 °C) and CO_2_. Spectra for each image pixel were obtained from the intensity values of the different detection channels by using the ImageJ software function “Plot Z-axis profile”^41^. The analysis was performed on manually selected regions of the plasma membrane which were not in contact with other cells (to avoid any effects of cell-to-cell contacts). The background signal was determined by applying selections of the same size on a dark region of the image, which was subsequently subtracted from the values obtained from the regions of interest. At least 24 regions of interest were analysed per condition for each independent experiment.

The average GP value for each selected region was calculated according to equation: 
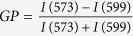
, where I(573) and I(599) are the fluorescence intensities at wavelengths 573 nm and 599 nm, respectively. The intensity values were directly obtained from the Ld membrane phases is arbitrary, the GP values are consistent only within one experimental study, and can not be directly compared to values obtained with other dyes or imaging settings. Therefore, a difference in GP value between different treatments and the ethanol control rather than the absolute GP value was used for the final quantification: 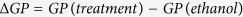.

### TIRF imaging

HEp-2 cells were seeded as for spectral GP imaging. The cells were washed twice with warm Live Cell Imaging Solution and then incubated with freshly prepared 10 nM NR12S solution in Live Cell Imaging medium for 7 min at 37 °C. The NR12S solution was replaced by fresh Live Cell Imaging medium and the first set of TIRF images was taken (time point 0 min). Then, 10 μM LPI or ethanol was added to the cells and a time-series of TIRF images was acquired starting at 5 min after the addition of the drugs. TIRF images were acquired using a DeltaVision OMX V4 system (Applied Precision, GE Healthcare) with a ring TIRF design (licensed to GE Healthcare from Yale University) rotating the laser beam at the back focal plane of the objective lens. The microscope was equipped with a 60x ApoN NA 1.49 objective (Olympus), 514 nm (100 mW diode) laser and three cooled sCMOS cameras (PCO). The stage and the objective were temperature controlled at 37 °C and the sample holder was enclosed by a humidified 5% CO_2_ incubation chamber. The system was controlled using the OMX Acquisition control software running under a Windows 7 operating system. Image registration was performed using the SoftWoRx (Applied Precision, GE Healthcare) software package running under a CentOS v6 (Linux) operating system. Two emission filters used, 541/22 and 609/37, corresponded to the regions of Lo and Ld phase of the NR12S spectrum, respectively. To visualise changes in GP values, pseudo-coloured GP images were generated using ImageJ macro adapted from Owen *et al*.^55^.

### Live cell imaging

HEp-2 cells were seeded in 35 mm MatTek glass bottom dishes at a density of 1 × 10^5^ cells/well one day before the experiment. At the day of the experiment, cells were washed once and incubated in serum-free HEPES-buffered cell growth medium and images were acquired on a DeltaVision microscope (Applied Precision, Issaquah, WA, USA) equipped with an Elite TruLight Illumination System, a CoolSNAP HQ2 camera and a 60x objective (Olympus, NA 1.42, Plan Apo N, UIS2, 1-U2B933). Images were captured under controlled CO_2_ conditions at 37 °C in a temperature-controlled incubation chamber. Image acquisition was started and 10 μM LPI or LPE was added after 10 min, and the imaging continued for 10 min. Brightfield illumination system with polarizer allowed us to obtain differential interference contrast images for whole cell morphology studies. Images were acquired every 60 sec for 20 min using softWoRx software (Applied Precision, GE Healthcare). Images illustrating the cell morphology before and after treatment were processed using Fiji software. To test potential toxic effects of LPI treatment, cells were left in the microscope chamber for 2 hours and control images were taken to ensure that cells had not become apoptotic or detached.

### Statistics

All experiments were performed in duplicates or triplicates. The experimental results are presented as mean values ± standard error of the mean (SEM) of *n* independent experiments, where *n* is indicated in each figure legend, if not otherwise stated. The data for presentation were normalized to control, with control set as 100% and two-tailed unequal variance *t*-test was used when treatment values were compared to normalized control. Two-tailed paired or unpaired *t*-tests were used to determine difference between means of two different treatments when appropriate. The levels of significance were set at *p ≤ 0.05, **p ≤ 0.01 and ***p ≤ 0.005.

## Additional Information

**How to cite this article**: Ailte, I. *et al*. Addition of lysophospholipids with large head groups to cells inhibits Shiga toxin binding. *Sci. Rep*. **6**, 30336; doi: 10.1038/srep30336 (2016).

## Supplementary Material

Supplementary Information

## Figures and Tables

**Figure 1:**
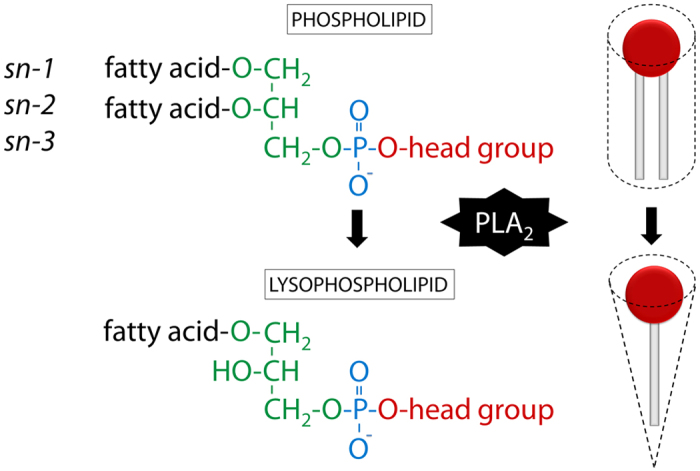
Phospholipid hydrolysis to lysophospholipid. Molecular structure and simplified drawing of a glycerophospholipid that is assumed to acquire a cylindrical shape and a lysophospholipid with a conical shape. The glycerol backbone is in green, the phosphodiester bond in blue and the head group in red. Structural nomenclature (*sn*) of carbons in the glycerol backbone is shown to the left and indicated by numbers from 1 to 3. Hydrolysis to lysophospholipid is catalysed by PLA_2_ in the *sn*-2 position.

**Table 1:** Origin and fatty acid chain composition of all lipids used in the study.

Lipid	Source organism/tissue	Manufacturer and cat. no.	Main species (%)			
			16:0	18:0	18:1	18:2
Edelfosine	synthetic	Tocris 3022		100		
LPA 18:0	synthetic	Avanti 857128		100		
LPC	chicken egg	Sigma-Aldrich L4129	66	33		
LPC 18:0	synthetic	Avanti 855775		100		
LPC 18:1	synthetic	Avanti 845875			100	
LPE	chicken egg	Avanti 860081	33.7	57	5.1	0.5
LPE (plasmalogen)	porcine brain	Avanti 850095			Predominant	
LPI 16:0	synthetic	Avanti 850102	100			
LPI 18:0	synthetic	Avanti 850104		100		
LPI	bovine liver	Avanti 850091	4	90	4	
LPS	porcine brain	Avanti 850092	1.1	90.1	4.1	

## References

[b1] Sandvig K., Bergan J., Kavaliauskiene S. & Skotland T. Lipid requirements for entry of protein toxins into cells. Prog. Lipid Res. 54, 1–13 (2014). 24462587 10.1016/j.plipres.2014.01.001

[b2] Marquardt D., Geier B. & Pabst G. Asymmetric lipid membranes: towards more realistic model systems. Membranes 5, 180–196 (2015). 25955841 10.3390/membranes5020180 PMC4496639

[b3] Sprong H., van der Sluijs P. & van Meer G. How proteins move lipids and lipids move proteins. Nat. Rev. Mol. Cell Biol. 2, 504–513 (2001). 11433364 10.1038/35080071

[b4] Coskun Ü. & Simons K. Cell membranes: the lipid perspective. Structure 19, 1543–1548 (2011). 22078554 10.1016/j.str.2011.10.010

[b5] Ingólfsson H. I. . Lipid organization of the plasma membrane. J. Am. Chem. Soc. 136, 14554–14559 (2014). 25229711 10.1021/ja507832e

[b6] Wilton D. C. In Biochemistry of Lipids, Lipoproteins and Membranes (Fifth Edition) (ed. Vance D. E. V. E.) 305–329 (Elsevier, 2008).

[b7] Lundbaek J. A. & Andersen O. S. Lysophospholipids modulate channel function by altering the mechanical properties of lipid bilayers. J. Gen. Physiol. 104, 645–673 (1994). 7530766 10.1085/jgp.104.4.645 PMC2229230

[b8] Shindou H., Hishikawa D., Harayama T., Eto M. & Shimizu T. Generation of membrane diversity by lysophospholipid acyltransferases. J. Biochem. (Tokyo) 154, 21–28 (2013). 23698096 10.1093/jb/mvt048

[b9] Tamura A., Tanaka T., Yamane T., Nasu R. & Fujii T. Quantitative studies on translocation and metabolic conversion of lysophosphatidylcholine incorporated into the membrane of intact human erythrocytes from the medium. J. Biochem. (Tokyo) 97, 353–359 (1985). 3997795 10.1093/oxfordjournals.jbchem.a135060

[b10] Wongkajornsilp A. & Rosenberry T. L. Uptake of exogenous sn-1-acyl-2-lyso-phosphatidylinositol into HeLa S3 cells. J. Biol. Chem. 270, 9147–9153 (1995). 7721829 10.1074/jbc.270.16.9147

[b11] Chernomordik L. Non-bilayer lipids and biological fusion intermediates. Chem. Phys. Lipids 81, 203–213 (1996). 8810049 10.1016/0009-3084(96)02583-2

[b12] McMahon H. T. & Boucrot E. Membrane curvature at a glance. J. Cell Sci. 128, 1065–1070 (2015). 25774051 10.1242/jcs.114454 PMC4359918

[b13] Corda D., Iurisci C. & Berrie C. P. Biological activities and metabolism of the lysophosphoinositides and glycerophosphoinositols. Biochim. Biophys. Acta BBA - Mol. Cell Biol. Lipids 1582, 52–69 (2002). 10.1016/s1388-1981(02)00137-3 12069810

[b14] Rantamäki A. H., Seppänen-Laakso T., Oresic M., Jauhiainen M. & Holopainen J. M. Human tear fluid lipidome: from composition to function. PLoS ONE 6, e19553 (2011). 21573170 10.1371/journal.pone.0019553 PMC3088682

[b15] Yamashita A. . Acyltransferases and transacylases that determine the fatty acid composition of glycerolipids and the metabolism of bioactive lipid mediators in mammalian cells and model organisms. Prog. Lipid Res. 53, 18–81 (2014). 24125941 10.1016/j.plipres.2013.10.001

[b16] Kihara Y., Maceyka M., Spiegel S. & Chun J. Lysophospholipid receptor nomenclature review: IUPHAR Review 8. Br. J. Pharmacol. 171, 3575–3594 (2014). 24602016 10.1111/bph.12678 PMC4128058

[b17] Grzelczyk A. & Gendaszewska-Darmach E. Novel bioactive glycerol-based lysophospholipids: new data - new insight into their function. Biochimie 95, 667–679 (2013). 23089136 10.1016/j.biochi.2012.10.009

[b18] Piñeiro R. & Falasca M. Lysophosphatidylinositol signalling: new wine from an old bottle. Biochim. Biophys. Acta BBA - Mol. Cell Biol. Lipids 1821, 694–705 (2012). 10.1016/j.bbalip.2012.01.009 22285325

[b19] Fang X. . Lysophospholipid growth factors in the initiation, progression, metastases, and management of ovarian cancer. Ann. N. Y. Acad. Sci. 905, 188–208 (2000). 10818454 10.1111/j.1749-6632.2000.tb06550.x

[b20] Ford L. A. . A role for L-α-lysophosphatidylinositol and GPR55 in the modulation of migration, orientation and polarization of human breast cancer cells. Br. J. Pharmacol. 160, 762–771 (2010). 20590578 10.1111/j.1476-5381.2010.00743.x PMC2931574

[b21] Liu Y. . Lysophosphatidic acid disrupts junctional integrity and epithelial cohesion in ovarian cancer cells. J. Oncol. 2012 (2012). 10.1155/2012/501492 PMC3346998 22593767

[b22] Ross R. A. L-α-lysophosphatidylinositol meets GPR55: a deadly relationship. Trends Pharmacol. Sci. 32, 265–269 (2011). 21367464 10.1016/j.tips.2011.01.005

[b23] Sutphen R. . Lysophospholipids are potential biomarkers of ovarian cancer. Cancer Epidemiol. Biomarkers Prev. 13, 1185–1191 (2004). 15247129

[b24] Mollinedo F. . Lipid raft-targeted therapy in multiple myeloma. Oncogene 29, 3748–3757 (2010). 20418917 10.1038/onc.2010.131

[b25] Escribá P. V. . Membrane lipid therapy: Modulation of the cell membrane composition and structure as a molecular base for drug discovery and new disease treatment. Prog. Lipid Res. 59, 38–53 (2015). 25969421 10.1016/j.plipres.2015.04.003

[b26] Bergan J. . The ether lipid precursor hexadecylglycerol protects against Shiga toxins. Cell. Mol. Life Sci. 71, 4285–4300 (2014). 24740796 10.1007/s00018-014-1624-1 PMC11113769

[b27] Flagler M. J., Mahajan S. S., Kulkarni A. A., Iyer S. S. & Weiss A. A. Comparison of binding platforms yields insights into receptor binding differences between Shiga toxins 1 and 2. Biochemistry (Mosc) 49, 1649–1657 (2010). 10.1021/bi902084y PMC2857392 20092352

[b28] Soltyk A. M. . A mutational analysis of the globotriaosylceramide-binding sites of Verotoxin VT1. J. Biol. Chem. 277, 5351–5359 (2002). 11723119 10.1074/jbc.M107472200

[b29] Kitov P. I. . Shiga-like toxins are neutralized by tailored multivalent carbohydrate ligands. Nature 403, 669–672 (2000). 10688205 10.1038/35001095

[b30] Kitova E. N. . Affinities of Shiga toxins 1 and 2 for univalent and oligovalent Pk-trisaccharide analogs measured by electrospray ionization mass spectrometry. Glycobiology 17, 1127–1137 (2007). 17686801 10.1093/glycob/cwm081

[b31] Jacobson J. M. . The crystal structure of Shiga toxin type 2 with bound disaccharide guides the design of a heterobifunctional toxin inhibitor. J. Biol. Chem. 289, 885–894 (2014). 24225957 10.1074/jbc.M113.518886 PMC3887212

[b32] Lee J. E. . Phylogenetic analysis of Shiga toxin 1 and Shiga toxin 2 genes associated with disease outbreaks. BMC Microbiol. 7, 109 (2007). 18053224 10.1186/1471-2180-7-109 PMC2211750

[b33] Bergan J., Dyve Lingelem A. B., Simm R., Skotland T. & Sandvig K. Shiga toxins. Toxicon 60, 1085–1107 (2012). 22960449 10.1016/j.toxicon.2012.07.016

[b34] Yamashita A. . The actions and metabolism of lysophosphatidylinositol, an endogenous agonist for GPR55. Prostaglandins Other Lipid Mediat. 107, 103–116 (2013). 23714700 10.1016/j.prostaglandins.2013.05.004

[b35] Thavarajah R., Mudimbaimannar V. K., Elizabeth J., Rao U. K. & Ranganathan K. Chemical and physical basics of routine formaldehyde fixation. J. Oral Maxillofac. Pathol. JOMFP 16, 400–405 (2012). 23248474 10.4103/0973-029X.102496 PMC3519217

[b36] Tanaka K. A. K. . Membrane molecules mobile even after chemical fixation. Nat. Methods 7, 865–866 (2010). 20881966 10.1038/nmeth.f.314

[b37] Bondarenko A. I., Malli R. & Graier W. F. The GPR55 agonist lysophosphatidylinositol directly activates intermediate-conductance Ca2+-activated K+ channels. Pflüg. Arch. - Eur. J. Physiol. 462, 245–255 (2011). 10.1007/s00424-011-0977-7 PMC3132407 21603896

[b38] Lingwood D. . Cholesterol modulates glycolipid conformation and receptor activity. Nat. Chem. Biol. 7, 260–262 (2011). 21460830 10.1038/nchembio.551

[b39] Yahi N., Aulas A. & Fantini J. How cholesterol constrains glycolipid conformation for optimal recognition of Alzheimer’s beta amyloid peptide (Abeta1-40). PloS One 5, e9079 (2010). 20140095 10.1371/journal.pone.0009079 PMC2816720

[b40] Beseničar M. P., Bavdek A., Kladnik A., Maček P. & Anderluh G. Kinetics of cholesterol extraction from lipid membranes by methyl-β-cyclodextrin—A surface plasmon resonance approach. Biochim. Biophys. Acta BBA - Biomembr. 1778, 17005–184 (2008). 10.1016/j.bbamem.2007.09.022 18068686

[b41] Schneider C. A., Rasband W. S. & Eliceiri K. W. NIH Image to ImageJ: 25 years of image analysis. Nat. Methods 9, 671–675 (2012). 22930834 10.1038/nmeth.2089 PMC5554542

[b42] Parasassi T., De Stasio G., d’Ubaldo A. & Gratton E. Phase fluctuation in phospholipid membranes revealed by Laurdan fluorescence. Biophys. J. 57, 1179–1186 (1990). 2393703 10.1016/S0006-3495(90)82637-0 PMC1280828

[b43] Sezgin E., Sadowski T. & Simons K. Measuring lipid packing of model and cellular membranes with environment sensitive probes. Langmuir 30, 8160–8166 (2014). 24905799 10.1021/la501226v

[b44] Kucherak O. A. . Switchable Nile Red-based probe for cholesterol and lipid order at the outer leaflet of biomembranes. J. Am. Chem. Soc. 132, 4907–4916 (2010). 20225874 10.1021/ja100351w

[b45] Darwich Z., Klymchenko A. S., Kucherak O. A., Richert L. & Mély Y. Detection of apoptosis through the lipid order of the outer plasma membrane leaflet. Biochim. Biophys. Acta BBA - Biomembr. 1818, 3048–3054 (2012). 10.1016/j.bbamem.2012.07.017 22846507

[b46] Mahfoud R., Manis A., Binnington B., Ackerley C. & Lingwood C. A. A major fraction of glycosphingolipids in model and cellular cholesterol-containing membranes is undetectable by their binding proteins. J. Biol. Chem. 285, 36049–36059 (2010). 20716521 10.1074/jbc.M110.110189 PMC2975227

[b47] Christiansson A., Kuypers F. A., Roelofsen B., Op den Kamp J. A. & van Deenen L. L. Lipid molecular shape affects erythrocyte morphology: a study involving replacement of native phosphatidylcholine with different species followed by treatment of cells with sphingomyelinase C or phospholipase A2. J. Cell Biol. 101, 1455–1462 (1985). 4044642 10.1083/jcb.101.4.1455 PMC2113896

[b48] Chowdhury H. H. . Lysophospholipids prevent binding of a cytolytic protein ostreolysin to cholesterol-enriched membrane domains. Toxicon 51, 1345–1356 (2008). 18455213 10.1016/j.toxicon.2008.03.010

[b49] Owen D. M., Williamson D., Rentero C. & Gaus K. Quantitative microscopy: protein dynamics and membrane organisation. Traffic Cph. Den. 10, 962–971 (2009). 10.1111/j.1600-0854.2009.00908.x 19416480

[b50] Bergmann W. L., Dressler V., Haest C. W. M. & Deuticke B. Reorientation rates and asymmetry of distribution of lysophospholipids between the inner and outer leaflet of the erythrocyte membrane. Biochim. Biophys. Acta BBA - Biomembr. 772, 328–336 (1984). 10.1016/0005-2736(84)90150-0 6722150

[b51] Mohandas N., Wyatt J., Mel S. F., Rossi M. E. & Shohet S. B. Lipid translocation across the human erythrocyte membrane. Regulatory factors. J. Biol. Chem. 257, 6537–6543 (1982). 7076680

[b52] Kavaliauskiene S. . Cell density-induced changes in lipid composition and intracellular trafficking. Cell. Mol. Life Sci. 71, 1097–1116 (2013). 23921715 10.1007/s00018-013-1441-y PMC11113877

[b53] Lingelem A. B. D., Hjelseth I. A., Simm R., Torgersen M. L. & Sandvig K. Geldanamycin Enhances Retrograde Transport of Shiga Toxin in HEp-2 Cells. PLoS ONE 10, e0129214 (2015). 26017782 10.1371/journal.pone.0129214 PMC4445914

[b54] Schindelin J. . Fiji: an open-source platform for biological-image analysis. Nat. Methods 9, 676–682 (2012). 22743772 10.1038/nmeth.2019 PMC3855844

[b55] Owen D. M., Rentero C., Magenau A., Abu-Siniyeh A. & Gaus K. Quantitative imaging of membrane lipid order in cells and organisms. Nat. Protoc. 7, 24–35 (2012). 22157973 10.1038/nprot.2011.419

