# Supplementary material for: Addition of lysophospholipids with large head groups to cells inhibits Shiga toxin binding
Source: Sci Rep. 2016 Jul 26;6:30336. doi: 10.1038/srep30336 (PMC4960542; doi:10.1038/srep30336)
Supplement: Supplementary Information [file srep30336-s1.doc]

# Supplementary information

# Addition of lysophospholipids with large head groups to cells inhibits Shiga toxin binding

Ieva Ailte^1,2,3^, Anne Berit Dyve Lingelem^1,2^, Simona Kavaliauskiene^1,2,3^, Jonas Bergan^1,2,4^, Audun Sverre Kvalvaag^1,2^, Anne-Grethe Myrann^1,2^, Tore Skotland^1,2^, Kirsten Sandvig^1,2,3*^

## Supplementary Figure S1: LPL treatment effect on Stx binding in HeLa cells


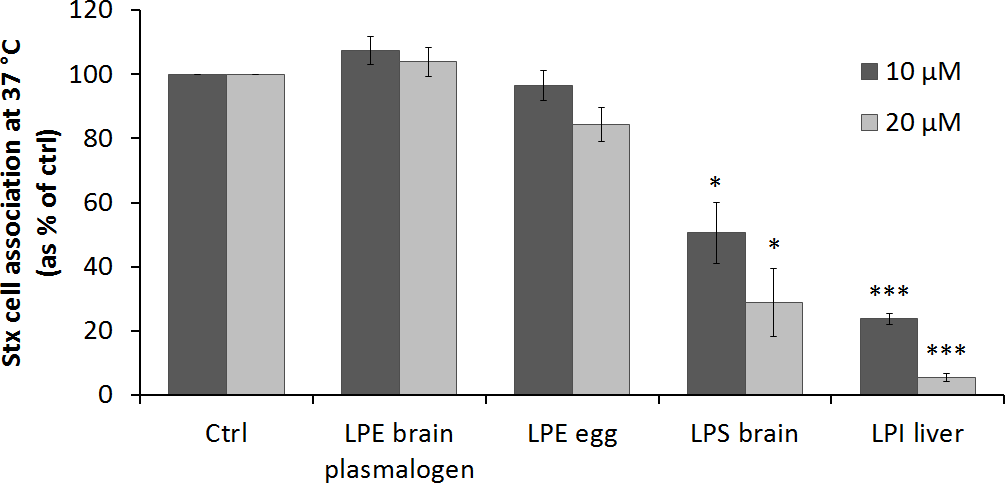


HeLa cells were pretreated with 10 or 20 µM LPLs (fatty acyl chains consisting of mostly C18:0) for 30 min in HEPES-buffered medium without FBS for 30 min at 37 °C, prior to incubation with ^125^I-Stx1m for 20 min at 37 °C. Cells were washed, lysed and the radioactivity was measured. Cell-associated ^125^I-Stx1m is expressed as % of control (mean ± SEM; *n* ≥ 3).
